# Comparison of Circulating Tumour Cells and Circulating Cell-Free Epstein-Barr Virus DNA in Patients with Nasopharyngeal Carcinoma Undergoing Radiotherapy

**DOI:** 10.1038/s41598-016-0006-3

**Published:** 2016-12-19

**Authors:** Jess Honganh Vo, Wen Long Nei, Min Hu, Wai Min Phyo, Fuqiang Wang, Kam Weng Fong, Terence Tan, Yoke Lim Soong, Shie Lee Cheah, Kiattisa Sommat, Huiyu Low, Belinda Ling, Johnson Ng, Wan Loo Tan, Kian Sing Chan, Lynette Oon, Jackie Y. Ying, Min-Han Tan

**Affiliations:** 1 Institute of Bioengineering and Nanotechnology, 31 Biopolis Way, #04-01, The Nanos, Singapore 138669, Singapore, Singapore; 2 Division of Radiation Oncology, National Cancer Centre Singapore, Singapore, Singapore; 3 JN Medsys Pte Ltd, Singapore, Singapore; 4 Department of Pathology, Singapore General Hospital, Singapore, Singapore; 5 Division of Medical Oncology, National Cancer Centre, Singapore, Singapore

## Abstract

Quantification of Epstein-Barr virus (EBV) cell-free DNA (cfDNA) is commonly used in clinical settings as a circulating biomarker in nasopharyngeal carcinoma (NPC), but there has been no comparison with circulating tumour cells (CTCs). Our study aims to compare the performance of CTC enumeration against EBV cfDNA quantitation through digital PCR (dPCR) and quantitative PCR. 74 plasma samples from 46 NPC patients at baseline and one month after radiotherapy with or without concurrent chemotherapy were analysed. CTCs were captured by microsieve technology and enumerated, while three different methods of EBV cfDNA quantification were applied, including an in-house qPCR assay for *Bam*HI-W fragment, a CE-IVD qPCR assay (S*entosa* ^®^) and a dPCR (Clarity™) assay for Epstein-Barr nuclear antigen 1 (*EBNA1*). EBV cfDNA quantitation by all workflows showed stronger correlation with clinical stage, radiological response and overall survival in comparison with CTC enumeration. The highest detection rate of EBV cfDNA in pre-treatment samples was seen with the *Bam*HI-W qPCR assay (89%), followed by *EBNA1*-dPCR (85%) and *EBNA1*-qPCR (67%) assays. Overall, we show that EBV cfDNA outperforms CTC enumeration in correlation with clinical outcomes of NPC patients undergoing treatment. Techniques such as dPCR and target selection of *Bam*HI-W may improve sensitivity for EBV cfDNA detection.

## Introduction

Nasopharyngeal carcinoma (NPC) is a malignant cancer of the nasopharynx, which is particularly common in parts of Southern China, South East Asia and North Africa^1^. Due to high rates of Epstein-Barr virus (EBV) nucleic acid detection in NPC, non-invasive approaches to diagnosis have focused on EBV as a target^2–4^. Post-treatment Epstein-Barr virus (EBV) cell-free DNA (cfDNA) levels have been demonstrated to correlate with NPC prognosis and recurrence^5 , 6^. EBV cfDNA can be quantified in the form of EBV single-copy genes; *EBNA1*, *LMP2* and *Pol-1*, or multiple-repeat fragments; *Bam*HI-W^7^. As there are six to twenty copies of *Bam*HI-W per EBV genome^8^, higher sensitivity is expected in *Bam*HI-W quantification assays. However, the variability of *Bam*HI-W copy numbers in different EBV isolates has been considered a challenges in assay comparison and standardization between laboratories^7 , 8^.

CTCs represent a circulating biomarker which has been extensively studied in many cancer types including breast, lung and colorectal cancer^9–12^. Due to challenges including platform costs and standardization, much less is known about CTCs in relatively neglected cancers such as NPC. There has been no previous comparison of performance and utility between circulating biomarkers such as CTCs and more conventional EBV cfDNA approaches, with only a comparison between different EBV DNA qPCR quantification assays involving different targets being previously reported^5^.

Hence, we investigate here the utility of various circulating biomarkers in NPC, with a special interest in the performance of CTC enumeration as a novel biomarker against more conventional EBV cfDNA quantitation using qPCR and digital PCR (dPCR) with *EBNA1* and *Bam*HI-W as targets.

## Results

### Comparison of sensitivity and specificity between EBV cfDNA assays

Benchmarking of the EBV cfDNA was conducted using comparison against results from a College of American Pathologists (CAP)-accredited laboratory as well as WHO-approved international EBV standards.

The clinical sensitivity and specificity of the three EBV cfDNA assays was benchmarked against an in-house EBV cfDNA assay targeting *EBNA1* in a College of American Pathologists (CAP)-accredited clinical-grade laboratory at the Singapore General Hospital (SGH), with known analytical performance reported as a sensitivity of 79% and specificity of 100%. With this assay, 46 NPC patients (Table 1), 31 (69%) were reported EBV-positive, 14 (31%) EBV-negative (1 case was not done due to logistic reasons). Of 31 EBV-positive patients on the clinical-grade assay, both *Bam*HI-W qPCR and *EBNA1*-dPCR assays showed 100% matching positivity, whereas the *EBNA1*-qPCR assay showed 80% match. Of the 14 EBV-negative patients, the *Bam*HI-W qPCR, *EBNA1*-dPCR and *EBNA1*-qPCR assay reported 9, 7, and 5 positive cases. Overall, all three EBV cfDNA assays demonstrate high clinical sensitivity and specificity, with particularly high sensitivity shown at baseline for the *Bam*HI-W qPCR assay, as expected.

**Table 1:** Patient characteristics.

Characteristic	No. of patients (%)
Total	46 (100.0)
Gender	
Male	38 (82.6)
Female	8 (17.4)
Age (median, 50; range, 23–80)	
≤50	24 (52.2)
>50	22 (47.8)
T-classification	
1	16 (34.8)
2	5 (10.9)
3	19 (41.3)
4	6 (13.0)
N-classification	
0	8 (17.4)
1	17 (37.0)
2	15 (32.6)
3	6 (13.0)
M-classification	
0	43 (93.5)
1	3 (6.5)
AJCC 7^th^ Stage	
I	8 (17.4)
II	8 (17.4)
III	18 (39.1)
IV	12 (26.1)
Treatment	
Radiotherapy alone	16 (34.8)
Chemo-Radiotherapy	27 (58.7)
Unknown	3 (6.5)
Adjuvant Chemotherapy	
Yes	3 (6.5)
No	43 (93.5)

Abbreviations: AJCC, American Joint Committee on Cancer.

The only available WHO-approved international EBV standard was used to benchmark the sensitivity and specificity of the three EBV cfDNA assays. The *Bam*HI-W qPCR assay demonstrated the highest reproducible sensitivity. The lowest EBV concentration detected in triplicates was 100 IU/mL for *Bam*HI-W qPCR assay and 1,000 IU/mL for both *EBNA1* assays (Table 2). The *Bam*HI-W qPCR assay was also able to detect positive signal in one replicate of the standard containing 1 IU/mL, whereas *EBNA1* assays were not able to. In addition, all assays produced no false-positive detection in five EBV-free standards, indicating their high specificity against EBV cfDNA.

**Table 2:** Sensitivity and Specificity of EBV cfDNA Quantitative Assays.

Spike-in Standards (IU/mL)	Total Number of Samples	Number of Positive Spike-in Standards		
		*Bam*HI-W qPCR Assay	*EBNA1*-qPCR Assay	*EBNA1*-dPCR Assay
1,000,000	3	3	3	3
1,000	3	3	3	3
100	3	3	1	2
10	3	2	1	0
1	3	1	0	0
0	3	0	0	0
Blank	2	0	0	0

Abbreviations: EBV, Epstein-Barr virus.

The IU of NIBSC standards is derived from a mean value of highly variable EBV copy number measured by various qPCR assays of 28 laboratories in the world^8^. These assays employ different DNA extraction methods, and target a wide range of genes, including a single-copy gene, *EBNA1*, and a multiple-repeat gene, *Bam*HI-W^8^. However, since dPCR was not included in the evaluation, the relationship between EBV copy number as obtained by dPCR and IU is less clear. Moreover, since the number of *Bam*HI-W fragments varies in different EBV isolates, a fixed conversion ratio of *Bam*HI-W copies to IU will not be always accurate in different patients’ sample. Therefore, the NIBSC standards were only used in this study for comparison of sensitivity and specificity between EBV cfDNA assays. The subsequent data were to be reported in copy number of respective EBV targets.

### Relationship between NPC circulating biomarkers in pre-treatment samples

Among EBV cfDNA quantitation approaches, *Bam*HI-W qPCR assay yielded the highest concentration of EBV cfDNA levels: 2.4 to 37.7-fold higher than *EBNA1*-qPCR assay and 2.2 to 25.5-fold higher than *EBNA1*-dPCR assay (Table 3). All samples detected EBV-positive by both *EBNA1* assays were also detected positive for EBV by *Bam*HI-W assay. The detection rates of canonical CTCs and potential CTCs are 76% and 94% in pre-treatment samples respectively. Overall, potential CTC count was higher and weakly correlated to canonical CTC count (r^2^ = 0.21, *P*-value = < 0.01). No correlation was observed between each type of CTC count and EBV cfDNA levels quantified by different assays. However, among the EBV cfDNA assays, strong correlation was observed between *Bam*HI-W qPCR and *EBNA1*-dPCR assays (r^2^ = 0.99, *P*-value < 0.0001), but not between *Bam*HI-W and *EBNA1*-qPCR assays (r^2^ = 0.03, *P*-value = 0.29) nor between *EBNA1*-qPCR and -dPCR assays (r^2^ = 0.06, *P*-value = 0.11). This result corresponded with the similar detection rate of *Bam*HI-W qPCR (89%) and *EBNA1*-dPCR (85%) assays, with the detection rate of *EBNA1*-qPCR assay being 67%.

**Table 3:** Quantitative levels of NPC circulating biomarkers in 46 pre-treatment samples.

Patient ID	AJCC 7^th^ Stage	Status on Follow-up	Pre-Treatment				
			*Bam*HI-W qPCR Assay (copies/mL)	*EBNA1*-qPCR Assay (copies/mL)	*EBNA1*-dPCR Assay (copies/mL)	Canonical CTCs (cells/mL)	Potential CTCs (cells/mL)
001	III	NED	70,569	5,416	9,484	0	3
002	III	NED	9,728	385	1,109	0	12
003	III	NED	10,631	855	1,376	NA	NA
004	III	NED	507	30	43	0	0
005	IV	NED	1,324	80	168	5	13
006	I	NED	0	0	0	4	13
007	I	NED	21	0	9	0	20
008	I	NED	107	0	0	0	14
009	III	DOD	18,572	5,425	3,636	NA	NA
010	IV	AWD	1,249	36	132	3	4
011	II	NED	23,507	2,838	3,656	2	1
012	IV	DOD	99,379	18,816	14,199	6	146
013	II	NED	301	0	28	0	0
014	I	NED	0	0	0	1	12
017	II	NED	67	0	21	50	134
018	IV	NED	441,316	13,565	50,081	11	29
019	I	NED	162	0	13	18	44
020	III	NED	4,860	0^a^	804	16	17
021	III	NED	1,236	33	49	37	76
022	III	NED	44,918	1,964	3,949	NA	NA
023	III	NED	29,006	777	3,272	NA	NA
024	I	NED	0	49	0	31	63
025	IV	NA	290,961	16,727	53,740	5	68
026	IV	NED	1,157	121	230	16	83
027	III	NED	6,687	431	1,356	4	21
028	I	NED	360	0	53	1	7
029	III	NED	6,072	303	816	17	47
030	III	NED	8,226	714	1,095	0	15
031	IV	NED	9,507	442	670	4	19
032	II	NED	92	0	42	3	67
033	IV	DOD	1,743,700	0^a^	193,125	6	44
034	III	NED	9,043	447	1,279	1	63
035	II	NED	146	21	0	1	14
036	III	NED	105	0	0	0	182
037	II	NED	669	0	63	3	13
038	IV	AWD	81	26	105	3	31
039	III	DOD	6,613	439	780	1	15
040	III	NED	5,106	623	1,125	1	14
041	II	NED	331	84	46	NA	NA
042	III	NED	2,156	171	241	NA	NA
043	III	NED	56,490	8,829	9,894	NA	NA
044	IV	DOD	88,432	12,074	16,850	NA	NA
045	IV	NED	33,057	6,014	5,319	NA	NA
046	I	NED	131	55	7	NA	NA
047	IV	NED	0	0	0	NA	NA
048	II	NED	0	0	7	NA	NA

Abbreviations: NPC, nasopharyngeal carcinoma; AJCC, American Joint Committee on Cancer; CTCs, circulating tumour cells; NED, no evidence of disease; AWD, alive with disease; DOD, dead of disease; NA, data are not available.

^a^PCR inhibition.

### Relationship between NPC circulating biomarkers and clinical stage

The clinical stages were re-classified to three groups; stage I, stage II-III, and stage IV (Table 4). The combination of stage-II and -III NPC patients was in the light of long-term 5-year follow-up data from Singapore showing similar survival outcomes using modern treatment approaches^13^. The EBV cfDNA levels in three assays strongly correlated with clinical stages. In contrast, there was no statistically significant relationship between CTCs and clinical stages. These results indicated a strong association between NPC clinical stage and EBV cfDNA, but not CTCs.

**Table 4:** Relationship between NPC circulating biomarkers and clinical stages in pre-treatment samples.

NPC circulating biomarkers	Mean Values			LR Chi-Square Values^a^	Degree of Freedom	*P*-Values^a^
	Stage I	Stage II–III	Stage IV			
*Bam*HI-W qPCR Assay (copies/mL)	98	12,140	225,847	14.15	1	0.0002^b^
*EBNA1*-qPCR Assay (copies/mL)	13	1,146	5,658	10.84	1	0.0010^b^
*EBNA1*-dPCR Assay (copies/mL)	10	1,699	27,885	14.52	1	0.0001^b^
Canonical CTC Enumeration (cells/mL)	8	8	7	0.05	1	0.8250
Potential CTC Enumeration (cells/mL)	25	39	49	1.07	1	0.3000

Abbreviations: NPC, nasopharyngeal carcinoma; CTCs, circulating tumour cells.

^a^Likelihood ratio Chi-square and *P*-values were determined using logistic ordinal regression for the prediction of NPC clinical stage, given the levels of NPC circulation biomarkers.

^b^ *P*-values < 0.05 were considered statistically significant.

### Relationship between NPC circulating biomarkers and treatment outcome

Decreased EBV cfDNA levels were observed in all EBV-positive patients following treatment, strongly correlating with the local radiological response (Table 5). To evaluate the predictive value of NPC circulating biomarkers for short-term radiological response, we determined that EBV cfDNA levels were significantly reduced after treatment (Wilcoxon’s signed rank testing p-value < 0.001 for all three techniques *Bam*HI-W qPCR, *EBNA1*-dPCR and *EBNA1*-qPCR assay). In contrast, for both canonical and potential CTCs, decrease was not significant (p = 0.07 and 0.54 respectively). The stratified analysis performed on patients undergoing radiotherapy and chemo-radiotherapy showed the magnitude of decrease of canonical CTCs pre- and post-treatment in each group remains insignificant (Supplementary Table 1). Overall, our results show that EBV cfDNA level correlation with short-term radiological response was much stronger than that of potential or canonical CTC counts.

**Table 5:** Quantitative levels of NPC circulating biomarkers in 28 matched samples.

Patient ID	AJCC 7^th^ Stage	Post-Treatment Radiological Response	Status on Follow-up	*Bam*HI-W qPCR Assay (copies/mL)		*EBNA1*-qPCR Assay (copies/mL)		*EBNA1*-dPCR Assay (copies/mL)		Canonical CTCs (cells/mL)		Potential CTCs (cells/mL)	
				Pre-Treatment	Post-Treatment	Pre-Treatment	Post-Treatment	Pre-Treatment	Post-Treatment	Pre-Treatment	Post-Treatment	Pre-Treatment	Post-Treatment
006	I	CR	NED	0	0	0	0	0	0	4	3	13	88
014	I	CR	NED	0	0	0	0	0	0	1	5	12	5
024	I	PR	NED	0	0	49	0	0	0	31	1	63	3
007	I	CR	NED	21	0	0	0	9	0	0	0	20	103
008	I	CR	NED	107	0	0	0	0	0	0	3	14	14
019	I	nCR	NED	162	42	0	0	13	0	18	22	44	148
028	I	CR	NED	360	0	0	0	53	14	1	0	7	13
017	II	PR	NED	67	0	0	0	21	7	50	16	134	220
032	II	PR	NED	92	0	0	0	42	7	3	0	67	15
035	II	nCR	NED	146	0	21	0	0	0	1	0	14	23
013^b^	II	CR	NED	301	0	0	0	28	0	0	3	0	26
011^b^	II	CR	NED	23,507	27	2,838	0	3,656	0	2	6	1	9
004^b^	III	CR	NED	507	0	30	0	43	0	0	4	0	26
021^b^	III	nCR	NED	1,236	0	33	0	49	0	37	2	76	33
029^b^	III	nCR	NED	6,072	0	303	0	816	0	17	0	47	6
027^b^	III	CR	NED	6,687	0	431	0	1,356	0	4	1	21	18
030^b^	III	nCR	NED	8,226	0	714	0	1,095	0	0	0	15	0
002^b^	III	nCR	NED	9,728	0	385	0	1,109	7	0	0	12	150
003^b^	III	CR	NED	10,631	0	855	0	1,376	0	NA	NA	NA	NA
009	III	PD	DOD	18,572	131	5,425	0	3,636	35	NA	NA	NA	NA
001^b^	III	CR	NED	70,569	0	5,416	0	9,484	0	NA	NA	NA	NA
023^b^	III	CR	NED	29,006	47	777	0	3,272	6	NA	NA	NA	NA
022^b^	III	nCR	NED	44,918	0	1,964	0	3,949	13	NA	NA	NA	NA
026^b^	IV	PR	NED	1,157	0	121	0	230	7	16	2	83	6
010^b^	IV	PR	AWD	1,249	0	36	0	132	0	3	0	4	3
005^b^	IV	nCR	NED	1,324	0	80	0	168	0	5	1	13	27
012^b^	IV	PR	DOD	99,379	24,577	18,816	2,529	14,199	5,107	6	1	146	35
018^b^	IV	PR	NED	441,316	0	13,565	0	50,081	0	11	6	29	61
Mean				27,690	887	1852	90	3,386	186	9	3	36	45
*P*-Values^a^				<0.001		<0.007		<0.001		0.07		0.54	

Abbreviations: NPC, nasopharyngeal carcinoma; AJCC, American Joint Committee on Cancer; CTCs, circulating tumour cells; nCR, near complete response; CR, complete response; PR, partial response; PD, progressive disease; NED, no evidence of disease; AWD, alive with disease; DOD, dead of disease; NA, data are not available

^a^P-Values were calculated using the Wilcoxon’s signed rank testing and values < 0.05 were considered statistically significant.

^b^Patients undergoing chemo-radiotherapy.

### Relationship between NPC circulating biomarkers and overall survival

Survival analysis demonstrated that there was a stronger correlation between EBV cfDNA and overall survival, as compared to that between CTC counts and overall survival. All three EBV cfDNA techniques showed prognostic value on survival analysis: *Bam*HI-W qPCR, *EBNA1*-dPCR and *EBNA1*-qPCR assays yielded corresponding p-values of 0.03, 0.02 and 0.0002 by log-rank testing respectively, whereas canonical CTC and potential CTC counts were not associated with overall survival (p = 0.66 and 0.13 respectively). Kaplan-Meier plots are also shown for dichotomized biomarker variables (Supplementary Figure 4).

## Discussion

Non-invasive approaches of NPC diagnosis have been available for the past decade via the detection of immunoglobulin A antibody against EBV antigens in patients’ serum^14 , 15^. However, these techniques are inefficient in NPC prognosis and relapse prediction^16 , 17^. There is considerable ongoing research into EBV cfDNA in NPC patients for prediction of post-treatment outcomes^6 , 18 , 19^, and its role in selecting patients for additional adjuvant treatment following definitive therapy.

In our study, good correlation between EBV cfDNA and clinicopathologic outcomes was consistently demonstrated regardless of approach undertaken: *Bam*HI-W qPCR, *EBNA1*-qPCR or *EBNA1*-dPCR assays. Decreased EBV cfDNA levels are commonly observed in almost all patients undergoing treatment, corresponding generally to the short-term post-treatment radiological response, which is commonly a complete or near-complete response. Overall, our results demonstrated that EBV cfDNA yielded better results in comparison with CTC count as a circulating biomarker for NPC. Regardless of approach, cfDNA showed far stronger correlation with tumor stage, short-term radiological response as well as overall survival, in comparison with CTC counts.

The detection rate of the in-house *Bam*HI-W qPCR assay was 89%, which was similar to a separate study targeting the same *Bam*HI-W fragment^18^, reporting 96% positive detection in Hong Kong NPC patients. In comparison with clinically validated assays, the in-house *Bam*HI-W qPCR assay demonstrated better performance. The detection rate of the CE-IVD *EBNA1*-qPCR assay reported in this study was 67%, despite its claimed clinical sensitivity of 100%, based on 80 EBV-positive samples. Moreover, EBV positive cases reported by the *Bam*HI-W qPCR assay were matched with the ones reported by the SGH assay, which had clinical sensitivity of 79%.

Despite being a powerful tool in NPC prognosis, the quantification of EBV cfDNA faces challenges of standardization. The NIBSC standards, which are derived from whole EBV produced by B95-8 cells^8^ provide a consensus estimate of EBV IU, but are not ideal for standardization of *Bam*HI-W copy number. In addition, the NIBSC spike-in standards do not truly represent the NPC plasma samples. Naturally occurring cfDNA has a size of less than 181 bp in NPC plasma^20^ whereas DNA obtained from NIBSC was genomic DNA with a size of 170 kb^21^. The differences in DNA size influence the choice of DNA extraction kit, which in turn has meaningful impact on DNA recovery, and subsequently DNA quantification. Unlike *Bam*HI-W qPCR and *EBNA1*-dPCR assays, the *EBNA1*-qPCR assay was performed using the automatic *Sentosa*® system integrated with both nucleic acid extraction and EBV quantification. The QIAamp Circulating Nucleic Acid Kit (Qiagen) used in *Bam*HI-W qPCR and *EBNA1-*dPCR assays were both designed for extraction of fragmented cfDNA as short as 75 bp whereas *Sentosa*® SX Virus Total Nucleic Acid Kit v2.0 (Vela Diagnostics) used in *EBNA1*-qPCR assay was optimized for total viral DNA extraction. As the comparison of assay performance was conducted on samples undergoing different extraction methods, the performance differences between the two platform technologies, qPCR and dPCR, may also reflect differences in extraction. However, this caveat does not change the conclusion that EBV cfDNA quantification outperforms CTC quantification. The variation in efficiency of DNA extraction kits could explain why *EBNA1*-qPCR and *EBNA1*-dPCR assays target the same EBV single-gene *EBNA1*, and yet differ much in detection rate in NPC plasma samples. Another reason for the higher detection rate of *EBNA1*-dPCR assay could be the difference in quantification platform in which dPCR technology carries the advantage of being more sensitive. By targeting the multiple-repeat *Bam*HI-W fragments, the in-house *Bam*HI-W qPCR assay yielded the highest detection rate in NPC pre-treatment samples. It also yielded the highest sensitivity in measurement of NIBSC spike-in standards despite the possible DNA losses due to the DNA extraction method potentially not optimized to genomic DNA. On the other hand, regardless of being different in fundamental techniques of quantification and EBV targets, *Bam*HI-W qPCR and *EBNA1*-dPCR assays were strongly correlated in the measurement of EBV levels in pre-treatment samples. This correlation could possibly be aided by the same extraction process from which the cfDNA used in *Bam*HI-W qPCR and *EBNA1*-dPCR assays was extracted. Altogether, in our interpretation, the in-house and dPCR assays are more likely to quantify the true values of EBV cfDNA level in pre-treatment samples of NPC patients. Nevertheless, as the absolute values of EBV cfDNA levels in clinical samples are unknown, it cannot be readily concluded which of the three assays performed with better accuracy. Another factor affecting EBV cfDNA quantification was earlier reported to be the PCR master mix^7^. The harmonization study concluded higher consistency of EBV cfDNA quantification in commercially available Roche master mix after being compared with an in-house master mix, which was more prone to batch-to-batch variations. It is certainly possible that master-mix differences could also contribute to such variation in detection.

The evidence of EBV cfDNA existing in the form of short and freely-floating fragments in the plasma had led to a conclusion that they were released from apoptotic NPC cells^20 , 22, 23^. In other words, the NPC cells releasing EBV cfDNA lysed before they had the chance to enter the bloodstream. This phenomenon could explain the non-correlation between NPC CTC counts and EBV cfDNA levels measured by various assays.

Overall, our results are the first comparison between EBV cfDNA and CTC count, showing that EBV cfDNA is a better biomarker than CTC enumeration in NPC prognosis and prediction of treatment outcomes, and reveals heterogeneity between NPC circulating biomarkers at the molecular and cellular levels. Our study also demonstrated that by targeting the multiple-repeat *Bam*HI-W, higher detection rate and sensitivity were achieved. Further, we demonstrate that dPCR is useful as a detection method for EBV cfDNA, with potential advantages over qPCR.

## Methods

### Clinical samples

The study was approved by the Centralised Institutional Review Board, SingHealth (Reference number: 2013/354/B) and all methods were carried out in accordance with the approved guidelines. A total of 46 NPC patients, all of Asian ethnicity, who provided informed written consent, were recruited into the study between June 2013 and October 2014 (Table 1). 20 mL of blood was collected in EDTA tube (BD Biosciences) at baseline and one month after treatment. All stage-I and most of stage-II patients received only radiotherapy whereas most patients from stage III and IV received combined chemo-radiotherapy. Only 3 patients received adjuvant chemotherapy. A total of 28 matched serial samples, pre- and post-treatment, were collected. The post-treatment radiological response of all patients was based on their first magnetic resonance imaging/computed tomography scan after treatment (Table 5). The median follow-up was 18.7 months.

### Participating laboratories and clinic

Institute of Bioengineering and Nanotechnology (IBN) served as the centralised laboratory of the study (Supplementary Figure 1). Blood samples were collected from consenting NPC patients at National Cancer Centre Singapore, and sent to IBN within the same day of their visits within 4 hours. For each sample, whole blood was used for immediate CTC enumeration, and plasma was obtained, assigned blinded IDs and stored at −80 °C until further use. Each plasma assay had its individually optimized volumes. 250 µL of frozen plasma was distributed to Singapore General Hospital (SGH) where cfDNA extraction and quantification was performed using the *Sentosa*® SA EBV Quantitative PCR Test (Vela Diagnostics) following manufacturer’s requirements. At IBN, 1 mL of thawed plasma was used for cfDNA extraction of which half was quantified by the in-house *Bam*HI-W assay. The other half of the extracted cfDNA was sent to JN Medsys where cfDNA quantification was conducted using the Clarity^TM^ Digital PCR System (JN Medsys).

### *Bam*HI-W qPCR assay

50 μL of cfDNA was extracted from 1 mL of thawed plasma using the QIAamp Circulating Nucleic Acid Kit (Qiagen). The *Bam*HI-W7 primers (Sigma Aldrich) and dual-labelled *Bam*HI-W7 hydrolysis probe (Life Technologies) were designed for the amplification of a 143-bp region of *Bam*HI-W. Each 20-µL reaction consisted of 1x Taqman® Fast Advanced Master Mix (Life Technologies), 400 nM *Bam*HI-W7 primers (sense 5′-*AGATCTAAGGCCGGGAGAGG*-3′ and antisense 5′-*CGCCCATTCGCCTCTAAAGT*-3′), 100 nM *Bam*HI-W7 probe (5′-(6-FAM)*CTCTGGTAGTGATTTGGACCCGAAATCTG*(TAMRA)-3′) and 2 µL of DNA template, which was equivalent to 40 μl of plasma. Standard calibrators for *Bam*HI-W were generated with 8 dilutions of DNA derived from EBV-immortalised cell lines (See Supplemental Materials) ranging from 1 to 10^7^ *Bam*HI-W copies per reaction. qPCR was performed using the ViiA™ 7 Real-time PCR System (Life Technologies). Each run included patients’ cfDNA, standard calibrators, EBV-positive, -negative and no-template controls (NTCs). The reactions were run at 50 °C for 2 min, followed by 95 °C for 20 sec to activate Uracil N-Glycosylase (UNG) and AmpliTaq® Fast DNA Polymerase, respectively. Subsequently, the reactions underwent 40 two-step cycles of denaturation and annealing at 95 °C for 1 sec, and 60 °C for 20 sec, respectively. The *Bam*HI-W copy number was automatically calculated from ViiA™ 7 software based on the *Bam*HI-W standard calibrator of each run, with R² = 0.99, qPCR efficiency = 98–100%, m = (−3.315) − (−3.368). Initial optimization of the *Bam*HI-W assay was conducted by conventional PCR using EBV-positive C666-1 DNA (Supplementary Figure 3). *Bam*HI-W specificity for healthy controls has been previously determined to be high^2^ and testing of 30 healthy donors also showed no signal.

### *EBNA1*-qPCR assay

The *Sentosa*® SA EBV Quantitative PCR Test (Vela Diagnostics) was applied for quantification of EBV cfDNA with the aid of the integrated *Sentosa*® SX101 (Vela Diagnostics) and Rotor-Gene® Q MDx 5-plex HRM (Qiagen) instruments. 60 µL of DNA was automatically extracted from 200 µL of plasma using the *Sentosa*® SX Virus Total Nucleic Acid Kit v2.0 (Vela Diagnostics). 10 µL of purified DNA, equivalent to 33 µL of plasma was used for each reaction. The PCR master mix contained reagents and enzymes for the amplification of a 79-bp fragment of *EBNA1*, as well as a second set of primers/probes designed to detect EC3, a control for PCR inhibition and cfDNA extraction. The concentration of *EBNA1* was automatically calculated based on the imported standard curve, with R² = 0.99, qPCR efficiency = 98%, m = (−3.367). The clinical sensitivity and specificity of the assay was reported as 100% and 98.8% respectively.

### *EBNA1*-dPCR assay

The Clarity^TM^ Digital PCR System (JN Medsys) was used. The assay was designed to amplify a 118-bp fragment of *EBNA1*. Each 15-µL reaction consisted of 1X FastStart Essential DNA Probes Master (Roche), 200 nM *EBNA1* primers (sense 5′-*TCATCATCATCCGGGTCTCC*-3′ and antisense 5′-*GCTCACCATCTGGGCCAC*-3′), 200 nM probe (5′-(6-FAM)*CCTCCAGGTAGAAGGCCATTTTTCCACCCTGTAG*(IABKFQ)-3′) (Integrated DNA Technologies), 1X Clarity^TM^ JN Solution (JN Medsys), 0.15 U UNG (Roche) and 3 µL of plasma DNA or controls. The equivalent plasma volume per reaction was 60 µL. Each reaction mix was incubated at 40 °C for 10 min to allow UNG to degrade carry-over PCR products, followed by 95 °C for 10 min for UNG inactivation. The reaction mix was partitioned into approximately 10,000 individual reactions in the Clarity^TM^ Digital PCR tube-strip (JN Medsys). Thereafter, the tube-strips were stabilised for 2 min, sealed with 230 µL sealing fluid and subjected to thermal cycling using the following parameters: 1 cycle at 95 °C for 5 min, 40 cycles at 95 °C for 50 sec and 58 °C for 1.5 min. Afterward, the tube-strips were transferred to the Clarity^TM^ Reader (JN Medsys), which detected and quantified fluorescence signals from all partitions. Absolute copy number of *EBNA1* in each reaction was determined by the Clarity^TM^ Software (JN Medsys) after analysis of the ratio of positive partitions (i.e. those that contained amplified products) over the total number of partitions, using Poisson statistics.

### Determination of sensitivity and specificity of EBV cfDNA assays

All three EBV cfDNA assays were benchmarked against the EBV qPCR assay routinely performed by the College of American Pathologists (CAP)-certified laboratory in SGH. The clinical sensitivity and clinical specificity of the SGH assay was reported as 79% and 100% respectively, based on 66 untreated nasopharyngeal carcinoma patients and 30 normal volunteers. In addition, sensitivity and specificity of EBV cfDNA assays were benchmarked against the 1^st^ World Health Organization (WHO) International Standards for EBV, code 09/260; from National Institute for Biological Standards and Control (NIBSC). The NIBSC standards and nuclease-free water were spiked into EBV-free plasma to obtain 18 standards of 6 known EBV concentrations, ranging from 0 to 1,000,000 IU/mL. In addition, two aliquots of EBV-free plasma served as blank standards. The protocol of DNA extraction, sample distribution and EBV cfDNA assays of spike-in standards was identical to the one for clinical plasma samples.

### Enumeration of NPC CTCs

CTCs from 1 mL of whole blood were captured using the microsieve technology and enumerated with the aid of biomarker characterization as described previously^24 , 25^. The microsieve technology is a size-based method capable of isolating both epithelial and mesenchymal CTCs, unlike the affinity system, which only captures EpCAM-expressed CTCs. Cell counting, and image analysis were performed subject to sample availability, using the MetaMorph software (Molecular Devices) and manually verified by trained laboratory technicians. Cytokeratin-positive and CD45-negative nucleated cells were classified as canonical CTCs. Other nucleated cells that were negative for both cytokeratin and CD45 biomarkers were defined as potential CTCs. All nucleated cells with CD45-positive were classified as white blood cells (Supplementary Figure 2).

### Statistical analysis

Correlation study was carried out to correlate EBV levels amongst the NPC circulating biomarkers assays. Logistic ordinal regression modelling was used to evaluate pre-treatment circulating biomarker quantitation relative to the dependent variable of clinical stage. Wilcoxon’s signed-rank test with continuity correction (R.3.0.0) was conducted to compare paired pre and post-treatment levels of NPC circulating biomarkers. Correlation was performed using Microsoft Excel and the logistic ordinal regression model was performed using the “orm {rms}” library package in R. Alpha was set to 0.05 throughout. Survival analysis was performed using R 3.0.0 *survival* package to study survival distributions of continuous pre-treatment levels of NPC circulating biomarkers and overall survival (Table 3), using log-rank testing to determine significance at a threshold of 0.05. 1 patient (Patient-025) was omitted from survival analysis, as the patient sought follow-up elsewhere.

## Electronic supplementary material

Supplementary Information

## References

[1] American Cancer Society. Nasopharyngeal Cancer. Available at: http://www.cancer.org/acs/groups/cid/documents/webcontent/003124-pdf.pdf (Accessed: 15th May 2015).

[2] Chan KCA (2014). Plasma Epstein-Barr virus DNA as a biomarker for nasopharyngeal carcinoma. Chin. J. Cancer..

[3] Chan KCA (2013). Early detection of nasopharyngeal carcinoma by plasma Epstein-Barr virus DNA analysis in a surveillance program. Cancer.

[4] Buglione M (2012). Circulating tumour cells in locally advanced head and neck cancer: preliminary report about their possible role in predicting response to non-surgical treatment and survival. Eur. J. Cancer..

[5] Le QT (2005). A comparison study of different PCR assays in measuring circulating plasma epstein-barr virus DNA levels in patients with nasopharyngeal carcinoma. Clin. Cancer Res..

[6] Chan ATC (2002). Plasma Epstein-Barr virus DNA and residual disease after radiotherapy for undifferentiated nasopharyngeal carcinoma. J. Natl. Cancer Inst..

[7] Le QT (2013). An international collaboration to harmonize the quantitative plasma Epstein-Barr virus DNA assay for future biomarker-guided trials in nasopharyngeal carcinoma. Clin. Cancer Res..

[8] Fryer, J. F. *et al*. Collaborative study to evaluate the proposed 1st WHO international standard for Epstein-Barr virus (EBV) for nucleic acid amplification technology (NAT)-based assays. *WHO* (2011).

[9] Saucedo-Zeni N (2012). A novel method for the *in vivo* isolation of circulating tumor cells from peripheral blood of cancer patients using a functionalized and structured medical wire. Int. J. Oncol..

[10] Krebs MG (2011). Evaluation and prognostic significance of circulating tumor cells in patients with non-small-cell lung cancer. J. Clin. Oncol..

[11] Aggarwal C (2012). Relationship among circulating tumor cells, CEA and overall survival in patients with metastatic colorectal cancer. Ann. Oncol..

[12] Bidard FC (2013). Clinical application of circulating tumor cells in breast cancer: overview of the current interventional trials. Cancer Metastasis Rev..

[13] Sommat, K. *et al.* Long term outcome of intensity modulated radiotherapy in nasopharyngeal carcinoma in National Cancer Centre Singapore. Paper presented at the 7th Biannual International Symposium on Nasopharyngeal Carcinoma, Yogyakarta, Indonesia. (2015).

[14] Neel HB, Pearson GR, Taylor WF (1984). Antibodies to Epstein-Barr virus in patients with nasopharyngeal carcinoma and in comparison groups. Ann. Otol. Rhinol. Laryngol..

[15] Uen WC, Luka J, Pearson GR (1988). Development of an enzyme-linked immunosorbent assay (ELISA) for detecting IgA antibodies to the Epstein-Barr virus. Int. J. Cancer..

[16] Neel HB, Pearson GR, Taylor WF (1984). Antibody-dependent cellular cytotoxicity. Relation to stage and disease course in North American patients with nasopharyngeal carcinoma. Arch. Otolaryngol..

[17] Neel HB, Taylor WF (1990). Epstein-Barr virus-related antibody. Changes in titers after therapy for nasopharyngeal carcinoma. Arch. Otolaryngol. Head Neck Surg..

[18] Lo YMD (1999). Quantitative analysis of cell-free Epstein-Barr virus DNA in plasma of patients with nasopharyngeal carcinoma. Cancer Research.

[19] Leung SF (2014). Plasma Epstein-Barr viral DNA load at midpoint of radiotherapy course predicts outcome in advanced-stage nasopharyngeal carcinoma. Ann. Oncol..

[20] Chan KCA (2003). Molecular characterization of circulating EBV DNA in the plasma of nasopharyngeal carcinoma and lymphoma patients. Cancer Res..

[21] Santpere G (2014). Genome-wide analysis of wild-type Epstein-Barr virus genomes derived from healthy individuals of the 1,000 Genomes Project. Genome Biol. Evol..

[22] Jahr S (2001). DNA fragments in the blood plasma of cancer patients: quantitations and evidence for their origin from apoptotic and necrotic cells. Cancer Res..

[23] Mutirangura A (1998). Epstein-Barr viral DNA in serum of patients with nasopharyngeal carcinoma. Clin. Cancer Res..

[24] Lim LS (2012). Microsieve lab-chip device for rapid enumeration and fluorescence *in situ* hybridization of circulating tumor cells. Lab Chip..

[25] Mohamed Suhaimi N-A (2015). Non-invasive sensitive detection of KRAS and BRAF mutation in circulating tumor cells of colorectal cancer patients. Mol. Oncol..

