# Supplementary material for: Comparison of Circulating Tumour Cells and Circulating Cell-Free Epstein-Barr Virus DNA in Patients with Nasopharyngeal Carcinoma Undergoing Radiotherapy
Source: Sci Rep. 2016 Dec 19;6:13. doi: 10.1038/s41598-016-0006-3 (PMC5431344; doi:10.1038/s41598-016-0006-3)
Supplement: Supplementary file 1 — Supplementary Information [file 41598_2016_6_MOESM1_ESM.pdf]

Comparison of Circulating Tumour Cells and Circulating Cell-Free Epstein-Barr Virus DNA in Patients with Nasopharyngeal Carcinoma Undergoing Radiotherapy

Jess Honganh Vo[1], Wen Long Nei[2], Min Hu[1], Wai Min Phyo[1], Fuqiang Wang[2], Kam Weng Fong[2], Terence Tan[2], Yoke Lim Soong[2], Shie Lee Cheah[2], Kiattisa Sommat[2], Huiyu Low[3], Belinda Ling[3], Johnson Ng[3], Wan Loo Tan[4], Kian Sing Chan[4], Lynette Oon[4], Jackie Y. Ying[1], Min-Han Tan[1,5*]

[1] Institute of Bioengineering and Nanotechnology, Singapore

[2] Division of Radiation Oncology, National Cancer Centre Singapore

[3] JN Medsys Pte Ltd, Singapore

[4] Department of Pathology, Singapore General Hospital

[5] Division of Medical Oncology, National Cancer Centre Singapore

Ms. Vo and Dr. Nei contributed equally to this work.

***Corresponding Author:** Dr. Min-Han Tan, Institute of Bioengineering and Nanotechnology, 31 Biopolis Way, The Nanos, Singapore 138669; Telephone: +65-68247110, Fax: +65-64789010; Email: mhtan@ibn.a-star.edu.sg

Supplemental Materials

Materials and Methods

Preparation of *Bam*HI-W standards Extracted DNA from EBV-immortalised cell lines was used for amplification of *Bam*HI-W region using *Bam*HI-W7 primers. The PCR products were purified by the NucleoSpin® Gel and PCR Clean-up (Macherey-Nagel). Electrophoresis gel was performed to confirm the purity of the PCR products**.** Purified PCR products were quantified using the QuantiFluor® dsDNA System (Promega) and the Quantus™ Fluorometer (Promega). Copy number of *Bam*HI-W was calculated using the formula below, with 88390.29 Da being the molecular weight of amplicon generated by *Bam*HI-W7 primers

$$\text{Copy Number of } BamHI - W = \frac{DNA\ Quantity\ (ng)\ x\ Avogradro's\ Number}{88390.29\ (\text{Da})\ x\ 10^9}$$

Ten-time serial dilution of PCR products was made to generate eight standards with the range between 0.5 to 5,000,000 copies per uL.

Results

Development and verification of *Bam*HI-W standards for IBN assay The PCR products generated by *Bam*HI-W7 primers were divided into three aliquots; of which, two were independently purified for removal of unwanted PCR components and one did not undergo the purification process. Electrophoresis gel showed single band at 143 bp in all aliquots (data not shown). Each aliquot was diluted to obtain equal *Bam*HI-W concentration of $5x10^6$ copies/ml and was amplified again with *Bam*HI-W7 primers. The results showed identical amplification curves in all aliquots (data not shown).

Supplementary Figure 1

Flow diagram of participants in the study

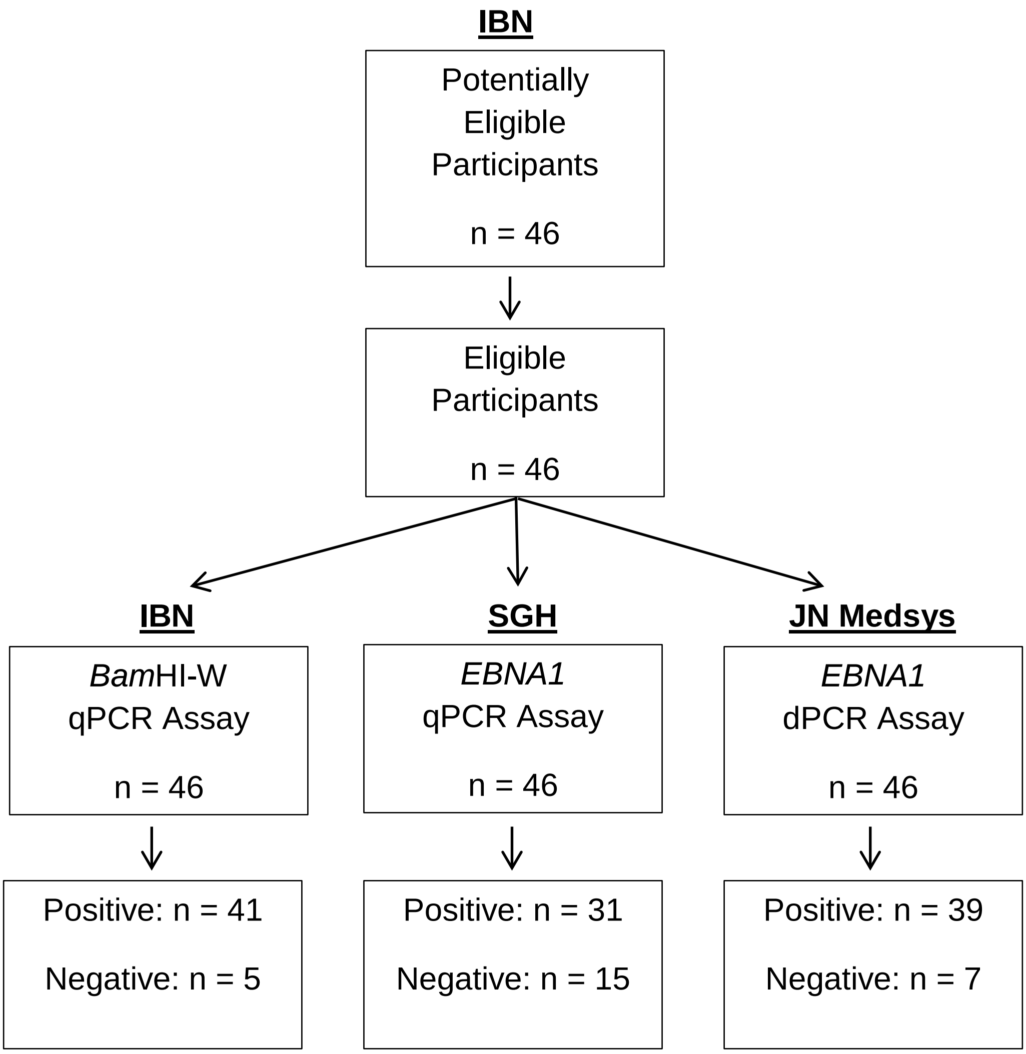

Supplementary Figure 2

Representative images of stained CTCs and white blood cells captured from 1 mL of whole blood of NPC patients. DAPI, 4',6-diamidino-2-phenylindole. Scale bars represent 20μm

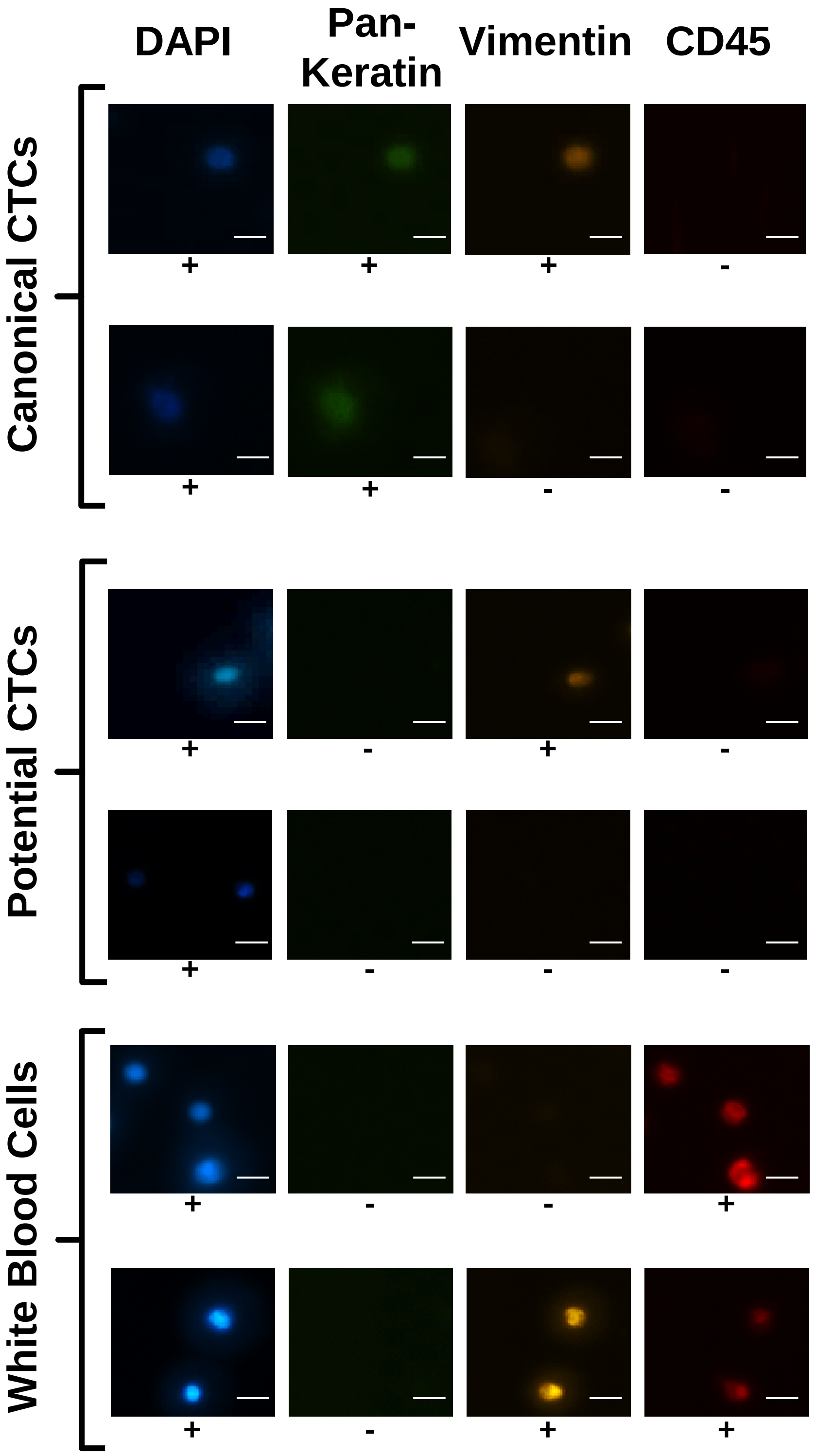

Supplementary Figure 3

Electrophoretic gel image of DNA products generated from 6 sets of *Bam*HI-W primers (1-6). 'a' denotes C666-1 (EBV-positive). 'b' denotes RKO (EBV-negative). Primer set number 3 was selected for the *Bam*HI-W qPCR assay.

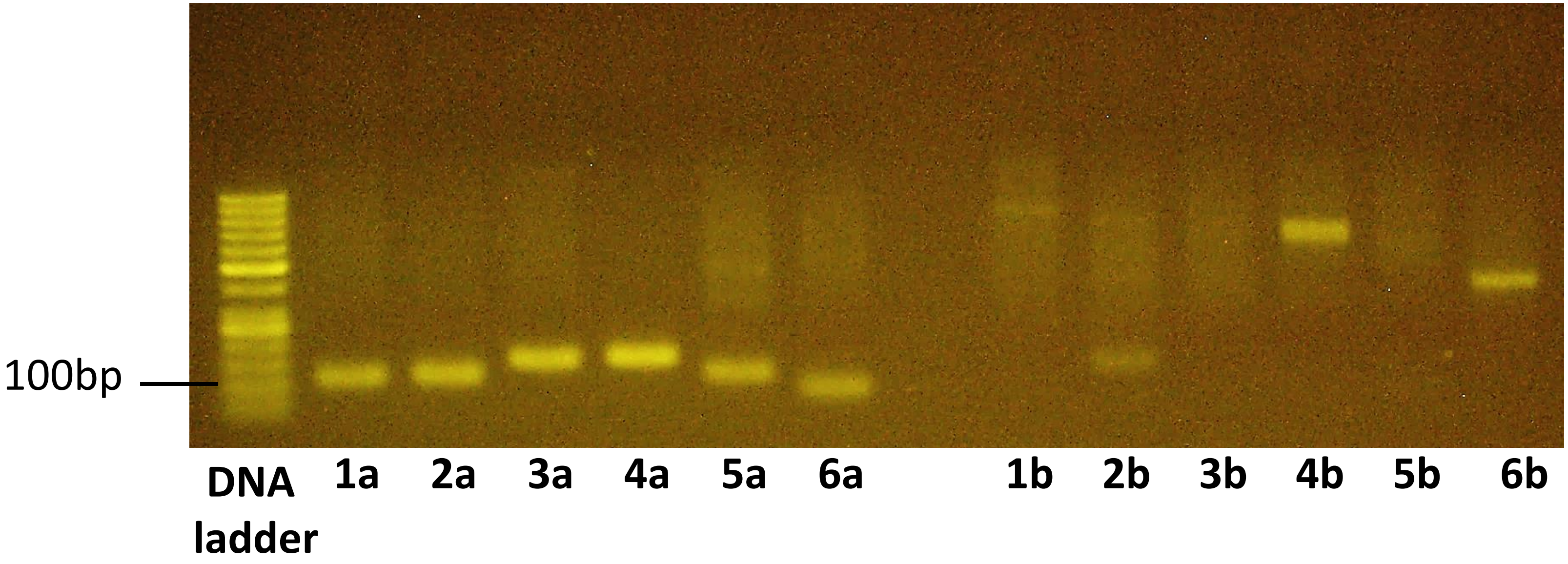

Supplementary Figure 4

Overall survival curves for NPC patients with different NPC circulating biomarker levels/counts measured by (A) *Bam*HI-W assay, (B) *EBNA1* qPCR-assay, (C) *EBNA1* dPCR-assay, (D) Enumeration of canonical CTCs, (E) Enumeration of potential CTCs. The numerical data were dichotomized based on the median of EBV cfDNA level or CTC counts

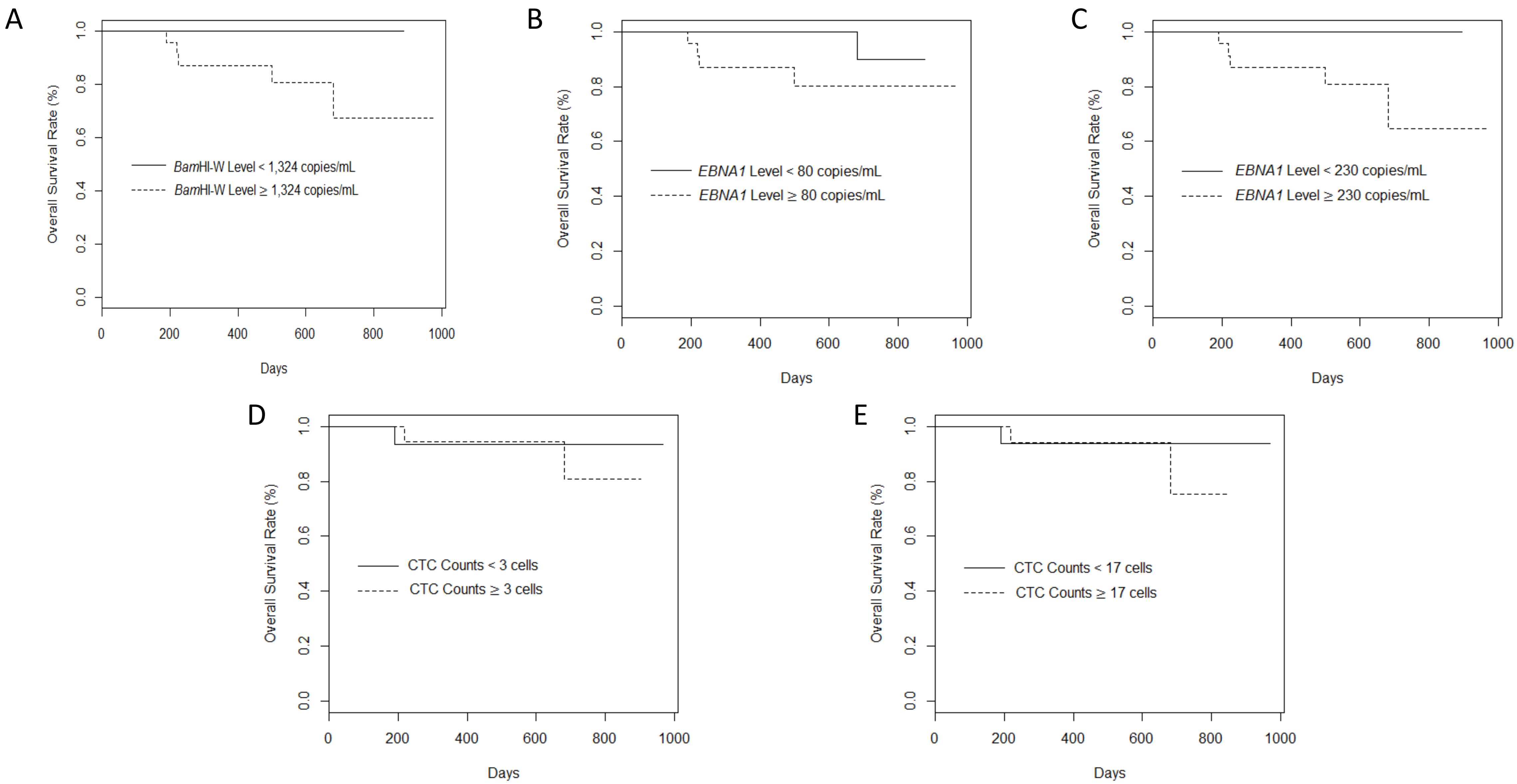

Supplementary Table 1. Stratified analysis of CTC enumeration in NPC pre- and post-treatment samples

	Radiotherapy (n=10)				Chemo-Radiotherapy (n=13)				All Treatment (n=23)			
	Canonical CTCs		Potential CTCs		Canonical CTCs		Potential CTCs		Canonical CTCs		Potential CTCs	
	Pre	Post	Pre	Post	Pre	Post	Pre	Post	Pre	Post	Pre	Post
**Mean**	11	5	39	63	8	2	34	31	9	3	36	45
**P-value**	0.59		0.19		0.07		0.78		0.07		0.54	
